# Optimization of Raw Ewes’ Milk High-Pressure Pre-Treatment for Improved Production of Raw Milk Cheese

**DOI:** 10.3390/foods11030435

**Published:** 2022-02-01

**Authors:** Rita S. Inácio, Rui Barros, Jorge A. Saraiva, Ana M. P. Gomes

**Affiliations:** 1 Department of Applied Technologies and Sciences, School of Agriculture, Polytechnic Institute of Beja, 7800-295 Beja, Portugal; rita.inacio@ipbeja.pt; 2 CBQF—Centro de Biotecnologia e Química Fina—Laboratório Associado, Escola Superior de Biotecnologia, Universidade Católica Portuguesa, Rua Diogo Botelho 1327, 4169-005 Porto, Portugal; rbarros@porto.ucp.pt; 3 LAQV-REQUIMTE—Laboratório Associado, Department of Chemistry, Campus Universitário de Santiago, University of Aveiro, 3810-193 Aveiro, Portugal

**Keywords:** design of experiment, *Serra da Estrela* cheese, microbial composition, yield, safety, high-pressure processing, experimental screening

## Abstract

Serra da Estrela protected designation of origin (PDO) cheese is manufactured with raw milk from Bordaleira and/or Churra Mondegueira da Serra da Estrela sheep breeds. Several socio-environmental shortcomings have reduced production capacity; hence, treatments that may contribute to its efficient transformation into cheese are welcome. High-pressure processing (HPP) milk pre-treatment may contribute to a cheese yield increment, yet optimization of processing conditions is warranted. An initial wide-scope screening experiment allowed for pinpointing pressure intensity, holding time under pressure and time after HPP as the most important factors influencing curd yield. Based on this, a more targeted screening experiment allowed for selecting the range of experimental conditions to be used for an experimental design study that revealed an HPP treatment at 121 MPa for 30 min as the optimum for milk processing to improve curd yield (>9%) and effectively maintain the beneficial cheese microbiota; the optimum was validated in a final experimental framework.

## 1. Introduction

In cheesemaking, the cheese yield (kg cheese/kg milk) is of particular economic interest since small differences in yield translate into big differences in both milk volume savings and final profits; the higher the solids percentage recovered, the greater the amount of cheese obtained, thus reflecting economic gains. In the particular case of the protected designation of origin (PDO) *Serra da Estrela* ewe cheese, the available milk is becoming scarcer due to limitations of various kinds, such as environmental and social cues. *Serra da Estrela* cheese is made solely with milk from Bordaleira *Serra da Estrela* and/or Churra Mondegueira ewe breeds, and according to specifications the milk cannot undergo any thermal treatment [1,2,3]. 

High-pressure processing (HPP) is a non-thermal food processing technology, wherein the food is subjected to a very high pressure range from 100–800 MPa during a holding period between 5–60 min. Different literature reports have indicated that HPP milk pre-treatment can increase curd yield [4,5]. Moreover, milk HPP has the potential to reduce viable cell numbers of undesirable contaminant micro-organisms, without significant effects on flavour and nutritional components, contributing to safer high-quality cheese products; however, HPP may influence the physicochemical and technological properties of the milk [6,7,8]. The effect of HPP on the curd yield has been evaluated mainly in cows’ milk [4,5,9,10,11] and goats’ milk [12,13,14], while only a few studies have focused on ewes’ milk [13,14]. In general, milk HPP pre-treatments have enabled an increase of the curd yield from about 4–23% in comparison to untreated milk. Huppertz et al. (2005) studied cow milk HPP pre-treatment between 100 and 600 MPa, having verified higher yield values (13–18%) at 100 and 250 MPa. On the other hand, one year before, the same group had verified lower values for HPP-treated milk at 250 MPa (exception for treatment for 60 min with 4% yield increasing) and higher values for HPP treatment at 400 and 600 MPa (4–23%) [5]. Furthermore, higher cow curd yield values were verified after maintaining the milk at 20 °C for 24 h post HPP treatment [5]. The same study revealed that a longer holding time under pressure (from 5 to 30 min) also increased the curd yield. In ewes’ milk, HPP treatments at 100 MPa for 30 min revealed a similar yield compared to untreated milk and an increase of about 5, 5 and 16% for 200, 300 and 400 MPa, respectively [13]. Ewes’ milk HPP treatment at 300 MPa with a holding time of 10, 20 and 30 min showed similar yield values but lower values when processing was for 5 min [13]. Similar results were verified in a further study by the same research group; López-Fandiño and Olano (1998) observed a higher yield after HPP at 40 °C than at 25 °C (about 23% vs. 9%) but reported that such treatment caused deleterious effects on gel firmness.

Several questions remain unanswered; hence, the main objectives of the current research were to use design of experiments (DoE) and response surface methodology (RSM) to determine the optimum HPP milk pre-treatment conditions that maximize curd yield while maintaining the beneficial microbiota of the cheese at most desirable levels for cheese biochemical properties development.

## 2. Materials and Methods

### 2.1. Screening Experimental Design and Rationale for Choice of Conditions 

**Figure 1:**
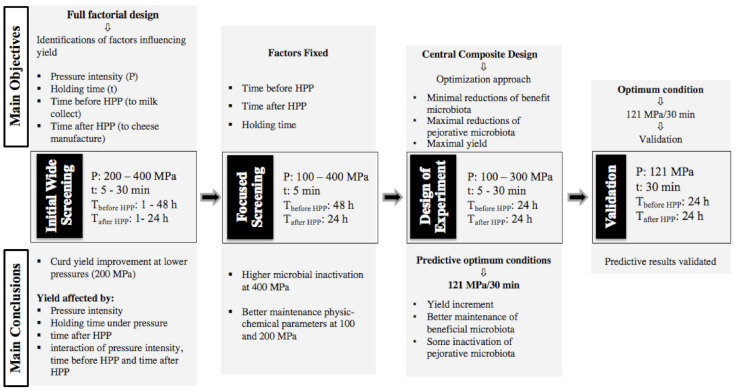
Schematic representation of initial wide screening, focused screening, design of experiment and validation.

Firstly, an initial wide screening study, two level full factorial design in triplicate for four factors (24 = 16*3 = 48 runs—see Table 1), was performed, in a randomized order, to identify the variables/factors with main effect and the interaction of factors on *Serra da Estrela* curd yield and time of coagulation. Based on previous studies in the literature, the four variables selected were: pressure intensity (range 200–400 MPa), holding time under pressure (range 5–60 min), time before HPP (1–48 h) and time after HPP (1–24 h), as shown in Figure 1. The waiting time before HPP and after HPP allowed for understanding if the storage time of milk prior to HPP and after HPP treatment influenced curd yield. The outcome parameters measured were yield and coagulation time. At the same time, untreated milk was studied as a control in order to compare with HPP-treated milk. 

Considering that the results of this initial wide screening study revealed more interesting results for lower pressures, 100 MPa was also studied, and the processing time was fixed at 5 min, because the processing time was found to have minor effect; further analyses were also carried out, namely pH, titratable acidity and microbiological enumeration. 

### 2.2. Surface Model—Optimization Experimental Design—Central Composite Design

Upon selection of the most important factors in the initial wide and the focused studies, an optimization design for curd yield improvement was established using a central composite design (Figure 1). This design consisted of a factorial design with two factors at two levels: pressure intensity ranged between 100–300 MPa and holding time under pressure between 5–30 min; additional axial and 5 central points were considered, as shown in Table 1, and dependent variables were technological cheese parameters (curd yield and coagulation time) and milk microbiota viable cell numbers (lactococci, lactobacilli, enterococci, *Enterobacteriaceae*, total coliforms, *E. coli*, staphylococci, yeasts and moulds counts).

### 2.3. Validation Experiment Design 

The theoretical optimum conditions 121 MPa/30 min (obtained for the optimization experiment design) were applied to raw ewes’ milk samples in a validation experiment in quintuplicate for greater validation robustness (untreated milk was also studied for data normalization).

### 2.4. Milk Supply

Raw ewes’ milk (from three farms in *Serra da Estrela* cheese PDO region, Portugal) was kept in a refrigerated tank until use, and prior to sampling milk was well mixed to ensure a homogeneous sample. Five litres of milk were used for the initial wide screening and another 5 L for the focused screening experiments, which were performed in December and January, respectively. For the response surface design, 8 L of milk was used in February and another 8 L in March for the model validation. 

### 2.5. Sample Packaging 

In the dairy, milk aliquots (≈75 mL) were placed into polyamide–polyethylene (PA–PE) bags (Plásticos Macar—Indústria de Plásticos Lda, Santo Tirso, Portugal) and heat sealed. The milk bags were stored under refrigeration (4 °C) before and after HPP treatment until analysis.

### 2.6. High-Pressure Processing

HPP was performed in 55 L capacity industrial scale high-pressure equipment (model 55, Hyperbaric, Burgos, Spain). For all experiments, the initial temperature of the water used as transmitting fluid was 8 °C. For the initial wide screening study: HPP was performed on the day of milk collection and after 48 h, as shown in Table 1 and Figure 1, the milk having been treated at 200 and 400 MPa for 5 and 30 min. For the focused screening study: the milk was treated after 48 h of collection and the curd transformation occurred after 24 h of HPP treatment, and milk samples were treated at 100, 200, 300 and 400 MPa for 5 min. For design of experiment, the milk was treated after 24 h of collection and the curd transformation occurred after 24 h of HPP treatment, according to Table 1. The validation step occurred using the optimum HPP conditions obtained in the design of experiment study, i.e., 121 MPa for 30 min. 

### 2.7. Yield and Coagulation Time 

Yield was estimated by centrifugation. Prewarmed milk (30 mL) to 32 °C was treated with 50 µL of standard vegetable rennet (*Cyanara cardunculus*, strength 1:15,000, Enzilab, Maia, Portugal). After 1 h at 32 °C, the curd was cut and 10 min later centrifuged at 1500× *g* for 15 min at 5 °C. The curd and whey were then separated and weighed. Coagulation time was evaluated by placing a spatula in the tubes every 10 min to see when the spatula came out of the curd free of any curd granules. 

### 2.8. Microbiological Analyses

Milk samples were added to and decimally diluted in 13.5 mL of sterile 0.1% (*w*/*v*) aqueous peptone and then plated, in triplicate, on several culture media. The following microbial groups were enumerated using the pour plate method: *Enterobacteriaceae* on violet red bile dextrose agar (VRBDA from Merck, Germany) and coliforms and *E. coli* on Chromocult coliform agar (CCA from Merck), both incubated at 37 °C for 1 d. The Miles and Misra technique [15] was used for enumeration of: total aerobic mesophilic micro-organisms on plate count agar (PCA from Merck) and incubated at 30 °C for 3 d; Enterococcus spp. on kanamycin aesculin azide agar base (KAAA from Oxoid, UK) and incubated at 37 °C for 1 d; Lactobacillus spp. on Man, Rogosa and Sharpe (MRS from Merck) and incubated at 30 °C for 3 d; Lactococcus spp. on M17 (Liofilchem, Roseto degli Abruzzi, Italy) and incubated at 30 °C for 3 d; Staphylococcus spp. on Baird-Parker agar (BPA from Merck) with egg yolk tellurite emulsion (Liofilchem) and incubated at 37 °C for 2 d; Listeria spp. on PALCAM agar selective agar base (Liofilchem), with selective supplement for PALCAM (Liofilchem) and incubated at 37 °C for 2 d; Pseudomonas spp. on pseudomonas agar base (PAB from Liofilchem) with glycerol and pseudomonas CFC supplement (CFC from Liofilchem) and incubated at 30 °C for 2 d. Petri dishes containing 10–100 colony forming units (cfu) were selected for counting. The results were converted into logarithmic decimals of the number of cfu per mL of milk. 

### 2.9. Physicochemical Analyses

The pH values of the milk and cheese were measured, at room temperature, in random points using a properly calibrated pH/temperature penetration pH meter (Testo 205, Testo, Inc., Sparta, NJ, USA). The titratable acidity was determined according to AOAC 947.05 [16] procedure for milk, using an automatic titrator with pH meter (Crison TitroMatic 1S with pH electrode 5.14, Barcelona, Spain), by titration to a pH value of 8.9. Physicochemical analyses were performed in triplicate per milk and cheese samples. 

### 2.10. Colour

Colour parameters were measured using a Minolta Konica CM-2300d (Konica Minolta CM-2300d, Osaka, Japan) at room temperature. The colour parameters were recorded in CIE Lab system and directly computed through the original SpectraMagic NX software (Konica Minolta, Osaka, Japan), according to the International Commission on Illumination regulations. Milk samples were kept 1 h at room temperature before measurements. Measurements were performed selecting six random spots, read in triplicate.

### 2.11. Statistical Analyses

For experimental design, Minitab and JMP software were used. SPSS software version 26.0 was used to evaluate the effect of factors and interactions in the initial wide screening study. For the focused screening, one-way analysis variance (ANOVA) was performed to establish the effect of different conditions (for HPP and untreated milk). The significant difference Tukey’s test was applied to compare the mean values of parameters, with the significance assigned at *p* < 0.05. 

## 3. Results and Discussion

### 3.1. Initial Wide Screening Study

In order to identify which factors may influence curd yield, a full factorial design was chosen where all possible combinations of all the input variables and their levels were included (Table 1 and Figure 1). Immediately after HPP treatment, the milk processed at 200 MPa was still liquid; however, the milk treated at 400 MPa revealed a more viscous texture, and after 24 h under refrigeration these samples revealed curd and whey separation. In the literature, a linear increase in skim milk viscosity was verified after HPP between 100 and 400 MPa for 30 min by Thom Huppertz, Fox and Kelly (2003). 

**Figure 2:**
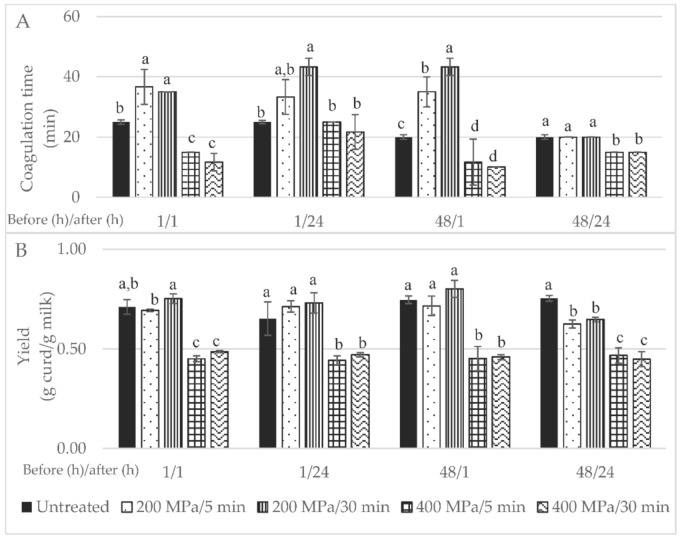
Initial wide screening results for: (**A**) coagulation time (in min) and (**B**) yield expressed in g of curd per g of milk; 1/1—milk high-pressure processing (HPP)-treated and transformed within the day of collection; 1/24—milk HPP-treated on day of collection and transformed the next day; 48/1—milk HPP-treated after 48 h of collection and subsequently transformed on the same day; 48/24—milk HPP-treated after 48 h of collection and transformed after 24 h; control samples are untreated milk. Different non-capital letters (a, b, c) indicate statistically significant differences for the same waiting time (*p* < 0.05) between treatment conditions.

In general, firmer curds were obtained from HPP processed milk for 5 min, while the milk treated for 30 min resulted in a curd/paste similar to a granular whey cheese (Figure S1). Rosina López-Fandiño, Mercedes Ramos and Olano (1997) also verified higher curd firmness for cow HPP-treated milk for 10 rather than for 30 min at 400 MPa. Results in the literature indicate that, for ewes’ milk, curd firmness was not affected by the HPP conditions (100–400 MPa for 30 min), while for goats’ milk firmness increased at 300 and 400 MPa [13]. In the present study, control samples revealed intermediate firmness compared to the HPP-treated samples. Faster coagulation occurred in HPP-treated milk at 400 MPa, particularly for a 30 min holding time (Figure 2A). The effect of HPP milk pre-treatment on coagulation time has been reported in the literature as being mainly dependent of pressure intensity and holding time. In this regard, a research group in this dominion treated bovine milk by HPP using different binomial pressure intensity/holding times [14,17,18]. These researchers were able to verify a reduction in coagulation time for HPP up to 200 MPa with treatment times within the range 10–60 min, while HPP treatment at 400 MPa only registered lower coagulation time when applied for 10 min; longer HPP holding times under such pressure increased coagulation time to values close to those of unprocessed milk. In a study performed with ovine milk, authors achieved results that support those presented in the current study; they were able to show that coagulation time decreased slightly after HPP 100 MPa for 30 min and increased significantly after HPP at 200–300 MPa to values 14–28% higher than untreated milk samples, albeit a new decrease for HPP at 400 MPa, although to values that remained slightly higher than those for untreated milk. Notably, gel firmness was not affected in the whole range of pressure studied (100–400 MPa) [13].

The timespan between HPP treatment and milk transformation into curd could also be a factor influencing coagulation time. In the present study, a lower coagulation time was verified when milk was transformed immediately into curd compared to curd production after 24 h storage of the HPP-treated milk under refrigeration (Figure 2). Zobrist, Huppertz, Uniacke, Fox and Kelly, (2005) [19] also reported a lower coagulation time for cows’ milk stored for short (after 0 and 4 h) than for long periods (after 24 and 48 h). This might be related to the fact that HPP leads to an increase in size and number of casein micelles, due to weakening of hydrophobic and electrostatic interactions between submicelles and further aggregation of submicelles to bigger clusters, with changes to form chains or clusters of submicelles ([6] cited in [20]).

Syneresis occurred only in those cheeses manufactured from HPP milk treated at the lower pressure under study (200 MPa) and also in control cheeses (see Figure S1). Low syneresis was reported in the literature for milk HPP-treated at higher intensity, e.g., treatments at 676 MPa/5 min at 10 °C for bovine milk [10], 600/15 min for skim milk [21], while treatments at 200 and 400 did not show significant differences [21].

Yield was improved by milk HPP pre-treatment at 200 MPa, as shown in Figure 2B; in particular, when the milk was treated for 30 min, after 48 h of refrigeration upon collection and transformation 1 h after HPP, a 12% increase in yield was achieved (*p* > 0.05). In contrast to what has been reported in the literature [5,13], in this study the milk HPP treatment at higher pressure intensity, i.e., 400 MPa, led to lower curd yields. Huppertz et al. (2004) reported a higher yield for cheeses made from HPP-treated cows’ milk (100–400 MPa) upon storage for 24 h at 20 °C than those produced immediately after HPP milk treatment. Furthermore, a higher curd formation yield was obtained with ewes’ milk HPP-treated at 200 MPa/30 min than with the control milk (11%); nevertheless, a considerable increase in curd yield was achieved after milk was HPP-treated at 400 MPa/30 min (15.6%) [13]. A similar HPP treatment (400 MPa/30 min) on cows’ milk led to a curd yield increase of 20% [18]. The increase in curd/cheese yield may be due to greater moisture retention but also to the incorporation of some denatured ß-lactoglobulin [18]. 

Statistical analysis of the data showed that the curd yield was affected by the pressure intensity (*p* < 0.001), holding time under pressure (*p* < 0.05), time after HPP (*p* < 0.05) as single factors and by the interaction of pressure intensity time before HPP and time after HPP (*p* < 0.001). This first step, i.e., the initial wide screening design, was crucial to determine that the pressure intensity, holding time under pressure and time after HPP were the most important factors when curd yield increment was desired. To the best of our knowledge, this is the first research work where all four factors and their interactions were studied for milk; furthermore, only Zobrist et al. (2005) studied the effect of cows’ milk storage after HPP and prior to rennet addition on coagulation time, having also verified different coagulation times after different milk HPP storage times of 4, 24 and 48 h at 4 and 20 °C. 

Based on the results obtained in the wide screening study and discussed above, milk refrigerated storage before and after HPP treatment was fixed at 48 and 24 h, respectively, since the time before HPP showed no individual effects on curd yield.

### 3.2. Focused Screening Design

As mentioned above, the initial wide screening design revealed more interesting results in milk HPP pre-treated at low pressure intensity (200 MPa) than at high pressure intensity (400 MPa). Therefore, in order to rule out any possibility of lower pressures bringing on more favourable results, in the focused screening design, the range of pressure intensity was widened to include also 100 MPa, and additional analyses were also carried out, namely pH, titratable acidity and microbiological data. 

**Figure 3:**
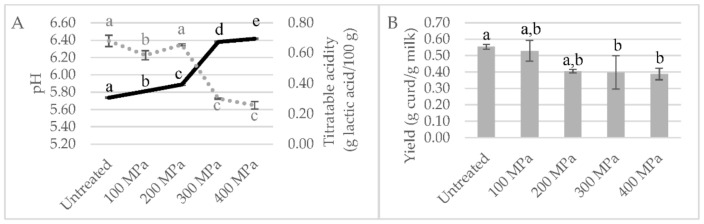
Focused screening study results: (**A**) milk pH values (continuous line) and titratable acidity (dots line); (**B**) yield expressed in g of curd per g of high-pressure processing (HPP)-treated milk at 100, 200, 300 and 400 MPa for 5 min after 48 h of collection and transformed after 24 h storage. Different non-capital letters (a, b, c, d, e) indicate statistically significant differences between the different conditions (*p* < 0.05).

As in the previous screening, HPP-treated milk at lower pressures, i.e., 100 and 200 MPa, remained in its liquid form, and the milk treated at 300 and 400 MPa became viscous and yellower and presented phase separation with time (Figure S2). This visual analysis is in agreement with the obtained curds (Figure S3) and milk pH values measured, as shown in Figure 3A. HPP-treated milk at 300 and 400 MPa revealed significantly higher pH values (6.38 and 6.42, respectively) than the control milk (5.74) (*p* < 0.001). Closer to the pH values of control milk were those of milk HPP-treated at 100 and 200 MPa (5.81 and 5.89), although statistically different (*p* < 0.001). In the literature, HPP goat milk treatment (500 MPa/15 min) led to significantly higher milk pH values in comparison to thermally pasteurized milk (6.66 vs. 6.54, respectively) [12]. Raw whole bovine milk revealed a similar effect, with HPP treatments (100, 250 and 400 MPa/15 min) inducing increments in milk pH values (to 6.73–6.75 vs. 6.66 of control milk) but without significant differences among HPP treatments [19]. These pH changes brought about by HPP can be due to dissolution of colloidal calcium phosphate (CCP), due to its dissociation from the casein micelle [22], possibly due to weakening of hydrophobic and electrostatic interactions between submicelles.

Titratable acidity was in agreement with the changes in pH values (Figure 3A). Relative to the curd yield, similar values were obtained for milk HPP-treated at 100 MPa and untreated milk (*p* > 0.05) (0.55 vs. 0.53 g milk/g curd), as shown in Figure 3B. 

As expected, HPP treatment at 400 MPa strongly affected microbial cell viability, in particular the beneficial microbiota that contribute positively to the cheese ripening process. On the other hand, many of the microbial groups tested, namely lactobacilli, enterococci, total mesophilic micro-organisms, staphylococci, coliforms and *Enterobacteriaceae* counts, were only slightly affected when milk was treated at 100 MPa (data not shown). Thus, a lower pressure intensity kept the beneficial microbiota and could improve the yield, but the minimization of spoilage bacteria such as staphylococci, coliforms and *Enterobacteriaceae* was not successfully achieved.

### 3.3. Optimization Design of Experiment by Central Composite Design

Based on the results obtained in the two screening studies, an optimization approach followed, where the factors to be studied included pressure intensity between 100 and 300 MPa, time of HPP treatment between 5 to 30 min (Table 1 and Figure 1), after 24 h of milk collection (note that this time period was reduced due to high viable cell numbers quantified in the focused screening design) and curd transformed after 24 h of HPP treatment.

**Figure 4:**
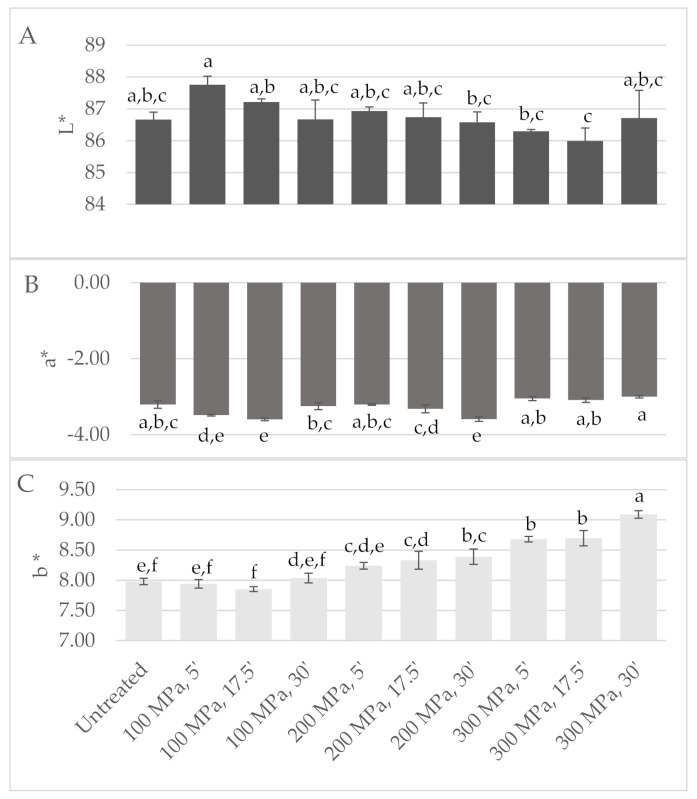
Design of experiment results: colour CIE (**A**) L*, (**B**) a* and (**C**) b* parameters measured in milk high-pressure processing (HPP) treated according to central composite design. Different non-capital letters (a, b, c, d, e, f) indicate statistically significant differences between the different conditions (*p* < 0.05).

Visual analysis of the milk bags upon treatment revealed that samples treated at 300 MPa for 5, 30 and 17.5 min (samples 3, 4 and 6, respectively, in Figure S4) were yellower. Instrumental colour analysis confirmed these colour variations, since these HPP-treated milks revealed higher b*-values (Figure 4); Gervilla, Ferragut and Guamis (2001) reported similar results for HPP-treated (100–300 MPa/15 min) raw ewes’ milk where an increase of b*-values was observed. Overall, HPP treatments at higher pressure intensity (200 and 300 MPa) led to a yellower milk colour (higher b*-values), particularly for longer holding times under pressure (17.5 and 30 min). Higher L*-values were measured for HPP-treated milk at 100 MPa for 5 and 17.5 min, while the other treatments led to similar or slightly lower L*-values than the control milk. The literature reports that HPP induces changes in L*-values mainly due to casein micelles disintegration into small fragments that increase the milk translucidity [23,24,25]. 

**Figure 5:**
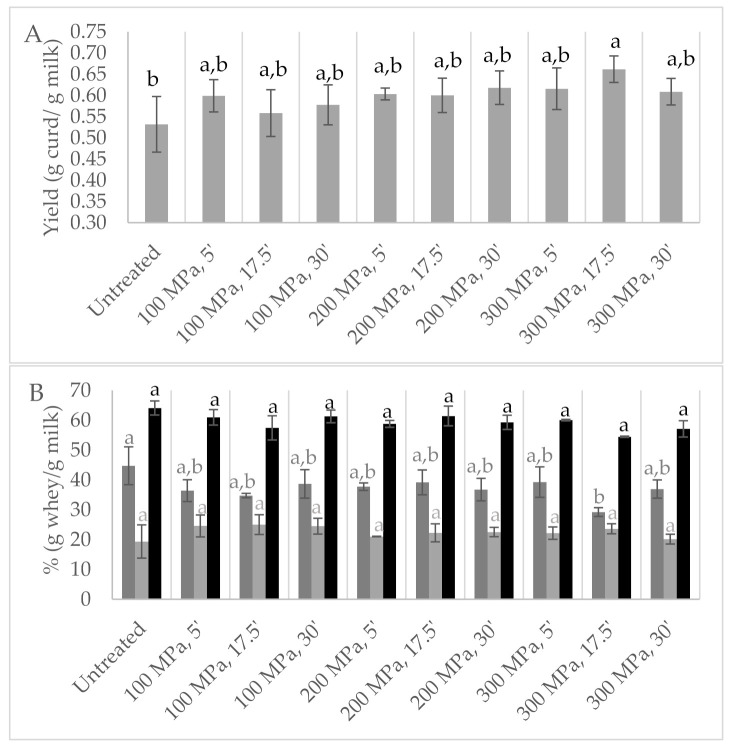
Design of experiment results: (**A**) yield expressed in g of curd per g of milk; (**B**) whey release immediately after centrifugation: first whey (■); syneresis (24 h) (■); whey + syneresis (■); from milk high-pressure processing (HPP)-treated according to central composite design. Control samples are untreated milk. Different non-capital letters (a, b) indicate statistically significant differences between the different conditions (*p* < 0.05).

HPP-treated milk resulted in curds (Figure S5) with increased yields between 5 and 24% in comparison to the control milk (Figure 5A), being the highest values achieved with milk treated at 300 MPa/17.5 min. To the best of our knowledge, there is only one work that studied HPP application on ewes’ milk, revealing a similar behaviour but reporting lower curd yields of about 5% for HPP-treated ewes’ milk at 200 and 300 MPa for 30 min, while at 100 MPa a yield similar to untreated milk was verified (10, 20 and 30 min of treatment time at 300 MPa showed no effect on yield) [13]. In the present study, the model analysis of the results revealed that the effect of the studied variables on yield could be described by a linear model, where pressure has the greatest contribution (*p* < 0.03 with lack of fit *p* = 0.067). 

Since during the two previous screening studies it was visually observed that syneresis showed a clearly different behaviour among samples, syneresis was also studied (Figure 5B). Initially after centrifugation, minor whey release was quantified for curds obtained from HPP milk pre-treatment, particularly for treatments at 100 and 300 MPa for 17.5 min (about 34 and 29%, respectively, against 45% for untreated milk), but syneresis after 24 h revealed lower, yet statistically insignificant, values for the control milk curds (*p* > 0.05). As previously mentioned, HPP may induce water retention in curd [11,26,27,28], a situation that appears to be related to a change in the structure of the *para*-caseinate network [26], an observation that may help explain the different syneresis behaviours observed for the HPP-treated samples. Relative to coagulation time, pre-treated HPP milk revealed at least 12% faster coagulation than untreated milk, as reported in the literature [13,14,17,19].

**Figure 6:**
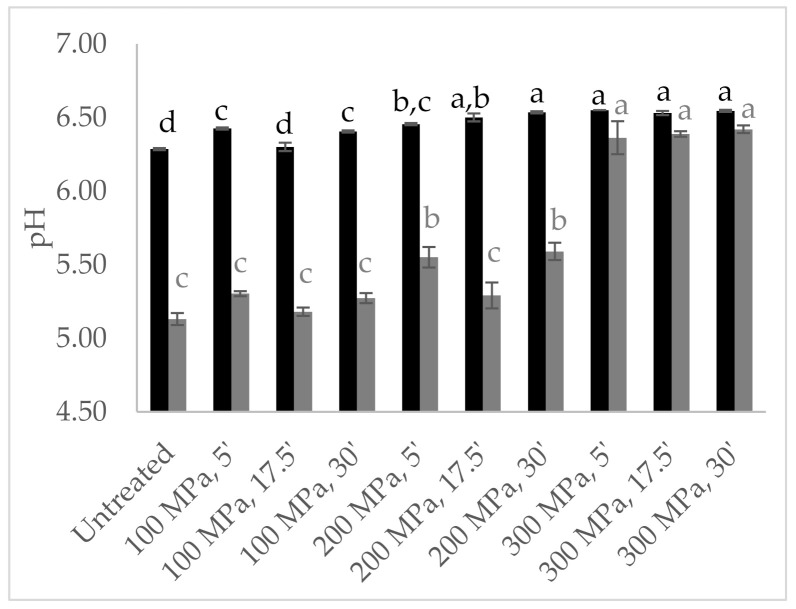
Design of experiment results: milk (■) and curd (■) pH values from milk high-pressure processing (HPP)-treated according to central composite design. Different non-capital letters (a, b, c, d) indicate statistically significant differences between the different conditions (*p* < 0.05).

The pH values were also analysed in untreated and HPP pre-treated milk and in the curds obtained therefrom, as shown in Figure 6. HPP-treated milk revealed higher pH values (6.4–6.5) than the untreated milk (6.29), a trend even more noticeable in milk pressurized at the highest pressure intensity (300 MPa), corroborating the results reported above, which can be justified by colloidal calcium phosphate solubilization [22]. Higher pH values were registered in curds resulting from HPP-treated milk (5.18–6.42) than untreated milk (5.13), being significantly higher in curd resulting from milk treated at 300 MPa (*p* < 0.001) (Figure 6). Similarly, about 0.6 pH units above that of the control cheese were reported for curd from HPP goat milk (400 MPa/5 min) [26]. However, higher intensity HPP treatments (586 MPa/1 min and 400–600 MPa/10 min) in bovine milk revealed no effect on curd pH [9] or led to a decrease [29].

**Figure 7:**
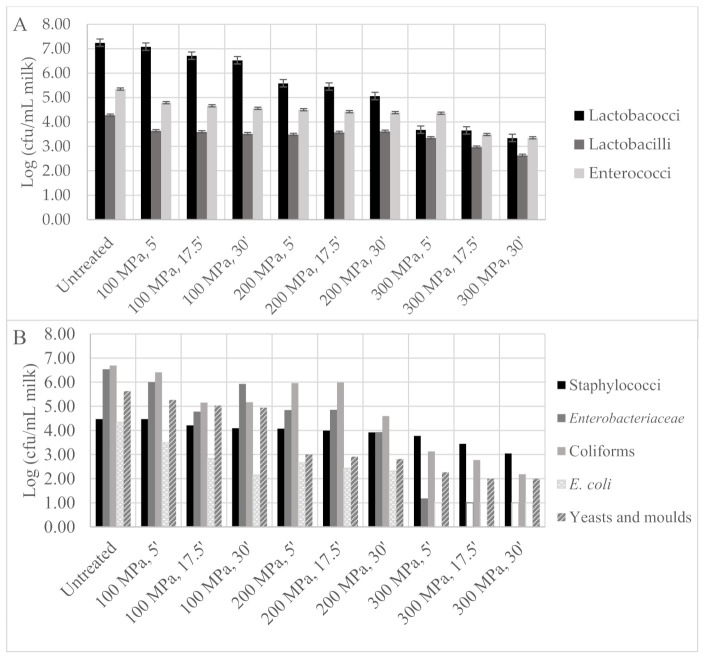
Design of experiment microbiota results: (**A**) lactobacilli, lactococci, enterococci and (**B**) staphylococci, *Enterobacteriaceae*, total coliforms, *Escherichia coli* and yeasts and moulds viable cell numbers in ewe milk samples after high-pressure processing (HPP) treatment according to central composite design. Empty bars represent microbial loads below the quantification limit (1.0 log cfu/mL).

Milk microbiota viable cell numbers are shown in Figure 7. In untreated milk samples, lactobacilli, lactococci and enterococci were found at 7.25, 4.28 and 5.35 log cfu/mL, respectively (Figure 7A). *Enterobacteriaceae* and total coliform viable cell numbers were found to be at a similar level, 6.53 and 6.69 log cfu/mL, respectively. *Escherichia coli* and *Staphylococcus* spp. were detected at 4.34 and 4.48 log cfu/mL, respectively. Yeasts and moulds were detected at 5.63 log cfu/mL (Figure 7B). As expected, a higher pressure intensity led to a higher microbial inactivation, particularly for longer holding times under pressure, as shown in Figure 7. Pressure lethal effect on micro-organisms was also reported to be higher as pressure increased from 100 to 300 MPa for 30 min in bovine milk [18], having the total aerobic counts achieved at an approximately 0.9 log reduction. 

HPP has been reported as an alternative to traditional thermal pasteurization in order to increase the microbial milk quality, but then lactic starter cultures needed to be added. This is an approach not possible for PDO cheeses, such as *Serra da Estrela* cheese, since the use of starter cultures is not allowed, and so a balance between spoilage microbiota inactivation while keeping as much as possible of the beneficial microbiota was necessary and became one of the objectives of the present work. Drake et al. (1997), Buffa, Guamis, Royo and Trujillo (2001) and Trujillo et al. (1999) treated bovine and goat milks at 586 MPa/1 min and 500 MPa/15 min, and the total viable cell numbers were reduced in 0.87–2.2 log cycles, coliforms in >1.3 log cycles and *Enterobacteriaceae* > 1.9–3.8 log cycles relativity to control milk. This same HPP treatment in goat milk led to lactobacilli reduction to below the quantification limit (>2.36 log cycles) [30]. 

The design of experiment results analysis allowed for optimization of the desirability answers (Table 2). In what concerns the microbiota, viable cell number reduction data were normalized by dividing by the mean of the microbial load of the untreated samples, expressing the microbial inactivation percentage. The model was then optimized in order to have: (1) the minimum values of normalized logarithmic reductions of lactobacilli, lactococci and enterococci (this family was added as a group to benefit, since its relevance in the development of cheese flavour is well known), (2) the maximum values of normalized logarithmic reductions of *Enterobacteriaceae*, total coliforms, *E. coli*, staphylococci and yeasts and moulds (known to be spoilage micro-organisms) and (3) the highest yield possible.

**Figure 8:**
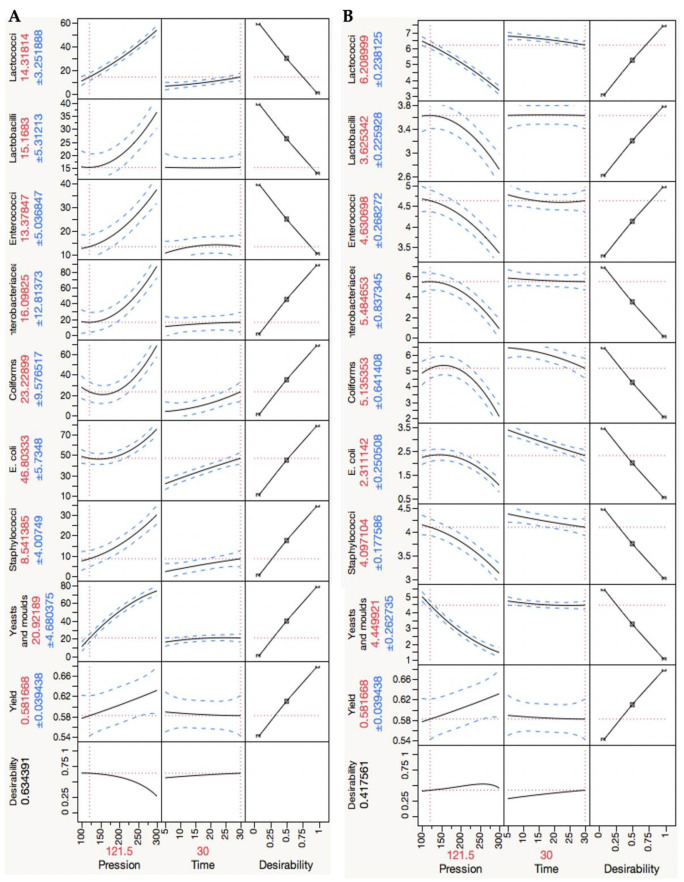
Design of experiment results: prediction profile of results optimization (by JMP9 software) of the surface model, assuming greater importance for lactobacilli and lactococci (quintuple of importance) and to enterococci (triple of importance). Equal importance was attributed to the remaining microbial groups/species. (**A**) Standard results are expressed in decimal reductions relative to the control load; (**B**) microbial viable cells numbers.

In this analysis, it was taken into account that not all the microbial groups have equal relevance to cheese maturation. Different importance levels were considered in the optimization design of experiment analysis to determine the optimal conditions, as shown in Table 2. An equal relevance attribution revealed 288.38 MPa for 5 min as the optimal HPP conditions. Considering the lactobacilli and lactococci values were fivefold more important, enterococci threefold more important and onefold for the other microbial groups under study, HPP treatment at 121.5 MPa for 30 min was achieved as the optimum conditions (the predicted results using these conditions are shown in Figure 8). 

### 3.4. Model Validation

The optimum condition obtained in the optimization study considering different importance levels, 121 MPa/30 min, was subsequently applied to new batch of raw ewes’ milk, in quintuplicate, for a greater robustness to validate the predicted results. Untreated milk was also studied to allow data normalization, and Table 3 and Figure S6 present all the obtained results: curd yield, microbiota viable cell numbers, whey quantification and pH values. Statistical analysis of all data obtained during model validation revealed that lactococci, lactobacilli, enterococci, *Enterobacteriaceae*, total coliforms and yeasts and moulds inactivation percentages (*p* > 0.05) were according to the predicted statistic prevision, thus validating these parameters (Table 3). However, *E. coli* and staphylococci inactivation was not validated (*p* < 0.05). Curd yield and released whey were also validated by the model (*p* > 0.05).

Thus, the HPP conditions 121 MPa for 30 min, when applied to raw ewes’ milk within 24 h after collection and transformed within the next 24 h to curd, were validated as the optimal conditions to combine the best possible inactivation of spoilage microbial viable cells, with a very low reduction of viable cell numbers of beneficial microbiota, and simultaneously achieve a better curd yield.

## 4. Conclusions

When screening the factors that affect curd yield, it is very important to test as many factors as possible in order to identify the significance of each of them. The experimental design allowed us to determine that the most influential factors on Serra da Estrela cheese production, from high-pressure-treated milk, were pressure intensity, holding time under pressure and time after HPP. A focused screening design was able to pinpoint that the viable cell numbers in milk HPP-treated at 400 MPa were considerably affected, while the lower pressure intensity kept the beneficial microbiota and improved the curd yield. For the identification of optima, a response surface design was performed, and higher pressure intensity led to a higher microbial inactivation, which was more pronounced at longer holding times under pressure. Nevertheless, placing as the main target an equilibrium between the best inactivation levels for spoilage bacteria without hindering (lowest reduction possible) beneficial microbiota viable cell numbers, coupled to an increased yield, led to determining HPP milk pre-treatment at 121 MPa for 30 min as the optimum condition, and model validation confirmed the predicted results. 

In conclusion, HPP treatment of raw ewes’ milk prior to cheese manufacture can enable *Serra da Estrela* curd yield increment and improve the microbial profile important from both safety and quality points of view.

## Figures and Tables

**Table 1:** Factors and levels for the initial wide screening and focused screening studies and optimization design of experiment (HPP stands for high-pressure processing).

Factors	Level (−1)	Level (0)	Level (+1)
**Wide screening study: two level full factorial design**			
Pressure intensity (MPa)	200		400
Holding time (min)	5		30
Before HPP time (h)	1		48
After HPP time (h)	1		24
**Focused screening study**			
Pressure intensity (MPa)	100		400
Holding time (min)	5		
Before HPP time (h)			48
After HPP time (h)			24
**Optimization design of Experiment: central composite design**			
Pressure intensity (MPa)	100	200	300
Holding time (min)	5	17.5	30
Before HPP time (h)		24	
After HPP time (h)			24

**Table 2:** Parameters considered in model optimization of the design of experiment (microbiota and yield) with response goals, with the respective different importance attributed and values expected to achieve by the modulation design at predicted optimized conditions.

	Response Goal	Importance	Expected Value	Importance	Expected Value	STD Adjusted
Lactococci	*Low*	1	45.4	25	14.2	1.38
Lactobacilli	*Low*	1	22.2	25	15.2	2.25
Enterococci	*Low*	1	20.1	15	13.4	2.13
*Enterobacteriaceae*	*High*	1	71.3	5	16.1	5.42
Coliforms	*High*	1	45.4	5	23.2	4.05
*Escherichia coli*	*High*	1	71.9	5	46.8	2.43
Staphylococci	*High*	1	15.1	5	8.5	1.70
Yeasts and moulds	*High*	1	61.1	5	20.9	1.98
Yield	*High*	1	0.62		0.58	
	Optimized conditions:	P (MPa)	Time (min)	P (MPa)	Time (min)	
		288.4	5	121.5	30	
	Desirability	0.59		0.61		

**Table 3:** Results for model validation: microbiota viable cell numbers were normalized and expressed in inactivation percentage (log reductions/log counts untreated milk samples). Prevision values and adjusted standard deviation determined by Minitab Software.

	Lactococci	Lactobacilli	Enterococci	*Enterobacteriaceae*	Coliforms	*Escherichia coli*	Staphylococci	Yeasts and Moulds
121 MPa, 30’ (A)	11.37	15.06	13.11	25.57	22.27	43.14	27.32	19.34
121 MPa, 30’ (B)	16.61	12.03	15.10	20.65	23.12	37.30	15.24	22.55
121 MPa, 30’ (C)	12.55	15.95	12.01	23.02	22.82	44.64	22.68	23.27
121 MPa, 30’ (D)	15.17	15.06	14.88	18.75	17.34	40.53	25.15	23.10
121 MPa, 30’ (E)	12.55	15.42	15.56	12.68	21.59	39.41	23.23	24.00
Prevision	14.23	15.17	13.38	16.10	23.22	46.80	8.54	20.92
STD (adjusted)	1.38	2.25	2.13	5.42	4.05	2.43	1.70	1.98
IC 95%	11.07	9.86	8.34	3.28	13.65	41.07	4.53	16.24
	17.57	20.48	18.42	28.91	32.80	52.54	12.55	25.60
	Validated	Validated	Validated	Validated	Validated	Not validated	Not validated	Validated
*p*-value	0.278	0.645	0.432	0.096	0.325	0	0	0.083

## Data Availability

Data is contained within the article.

## Supplementary Materials

The following supporting information can be downloaded at: https://www.mdpi.com/article/10.3390/foods11030435/s1, Figure S1. First screening: curds obtained from milk HPP at different conditions: (A) after centrifugation and whey separation, 0 h; (B) after 2 h; (C) after 24 h; Figure S2. Second screening: milk bags (A) 1 h after HPP at 100, 200, 300 and 400 MPa for 5 min; (B) after 24 h; Figure S3. Second screening: curds obtained from milk HPP at 100, 200, 300 and 400 MPa for 5 min (A) 0 h after centrifugation and (B) after 24 h; Figure S4. Surface Model: milk bags (A) before HPP treatment and (B) immediately after HPP treatments according to central composite design; Figure S5. Surface Model: curds obtained from HPP milk pre-treated according to central composite design (A) 0 h after centrifugation and (B) after 2 h; Figure S6. Validation model: (A) yield expressed in g of curd/g of milk; (B) whey release immediately after centrifugation: first whey; syneresis (24 h); whey + syneresis; (C) milk and curd pH values from HPP milk pre-treated.
