# Supplementary material for: Optimization of Raw Ewes’ Milk High-Pressure Pre-Treatment for Improved Production of Raw Milk Cheese
Source: Foods. 2022 Feb 1;11(3):435. doi: 10.3390/foods11030435 (PMC8834186; doi:10.3390/foods11030435)
Supplement: Supplementary file 1 [file foods-11-00435-s001.zip › foods-1557645-supplementary.pdf]

Supplementary Material

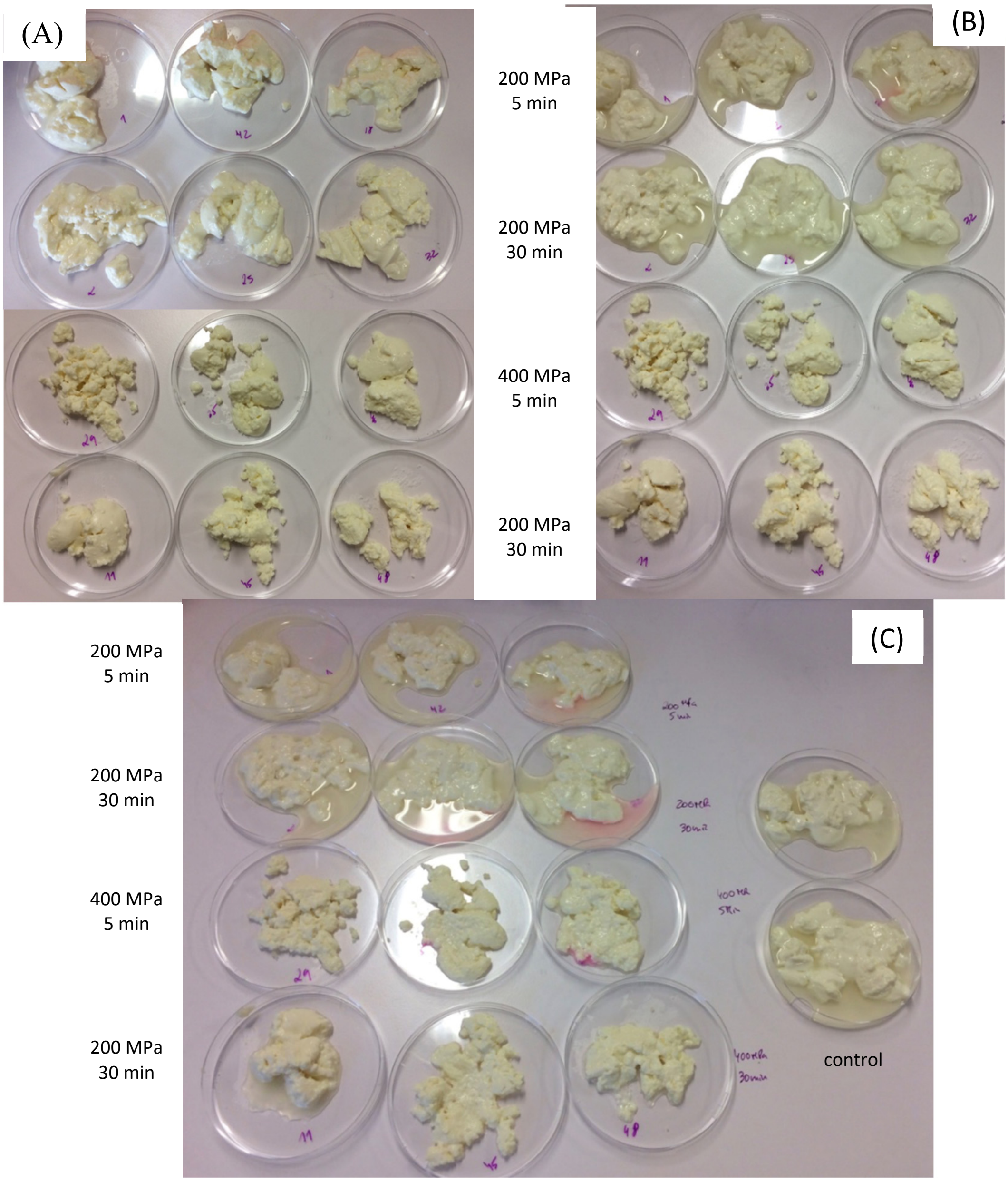

Figure S1. First screening: curds obtained from milk HPP at different conditions: (**A**) after centrifugation and whey separation – 0 h; (**B**) after 2 h; and (**C**) after 24 h.

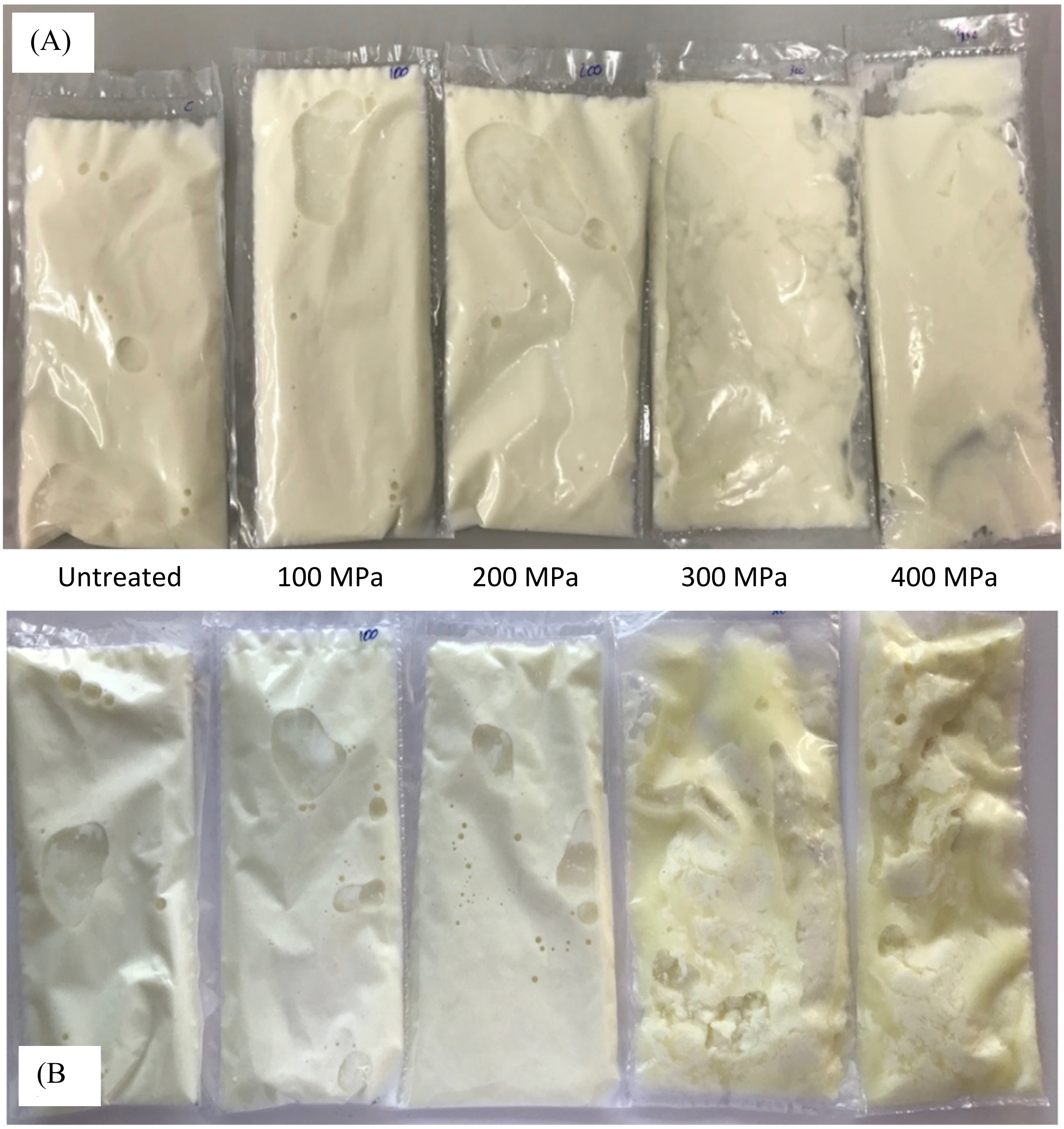

Figure S2. Second screening: milk bags (**A**) 1 h after HPP at 100, 200, 300 and 400 MPa for 5 min; (**B**) after 24 h.

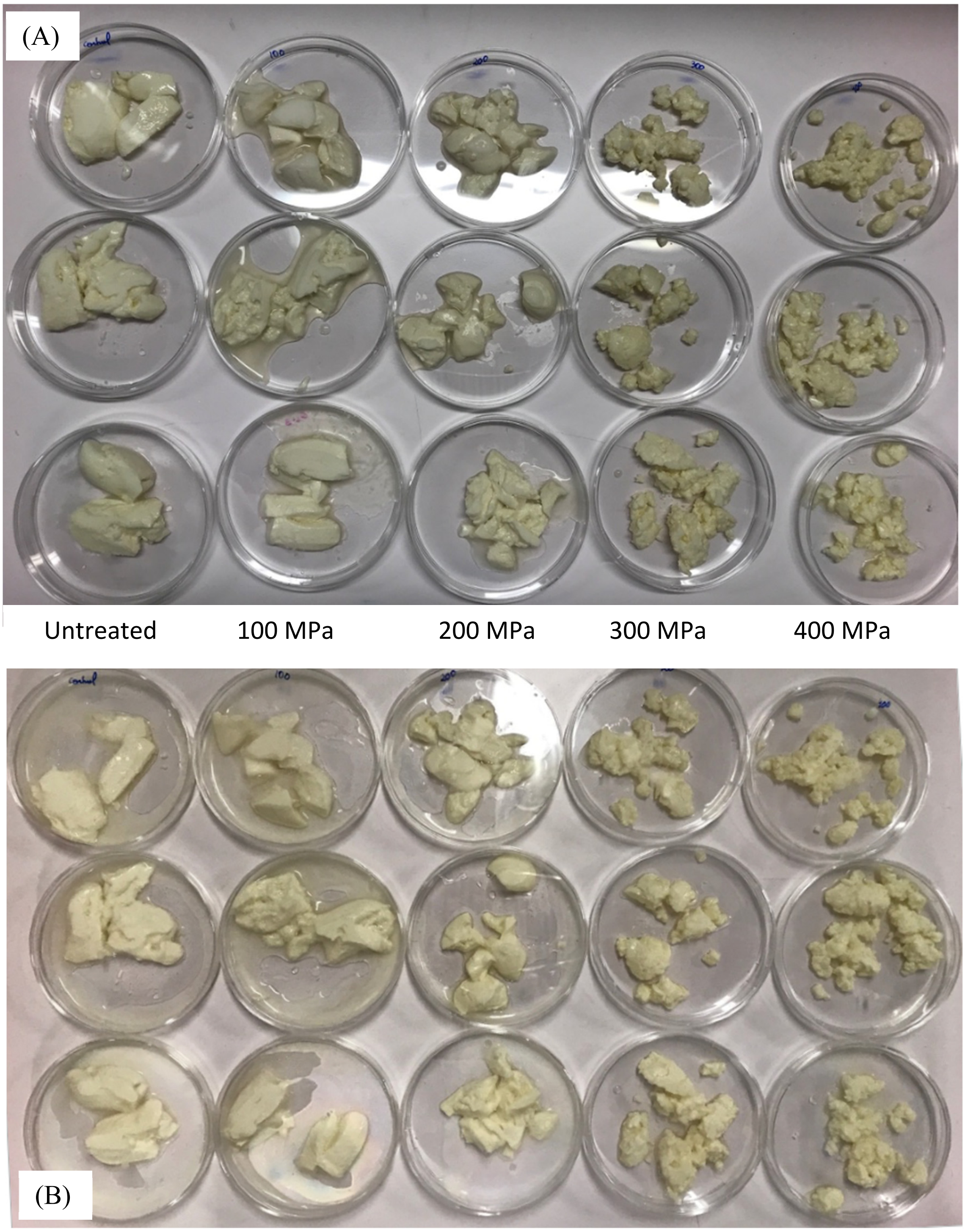

Figure S3. Second screening: curds obtained from milk HPP at 100, 200, 300 and 400 MPa for 5 min (**A**) 0 h after centrifugation and **(B**) after 24 h.

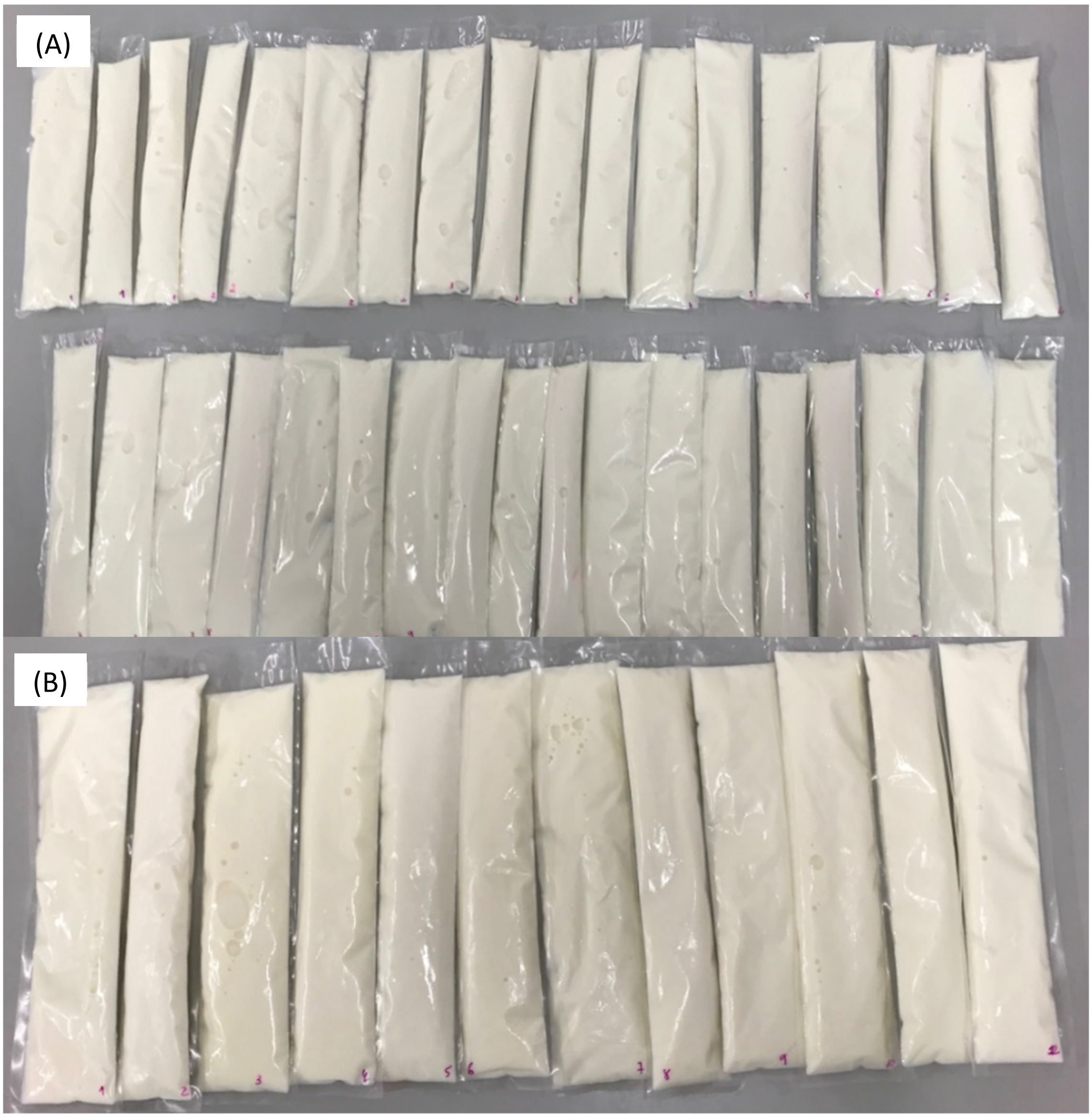

Figure S4. Surface Model: milk bags (**A**) before HPP treatment and (**B**) immediately after HPP treatments according to central composite design.

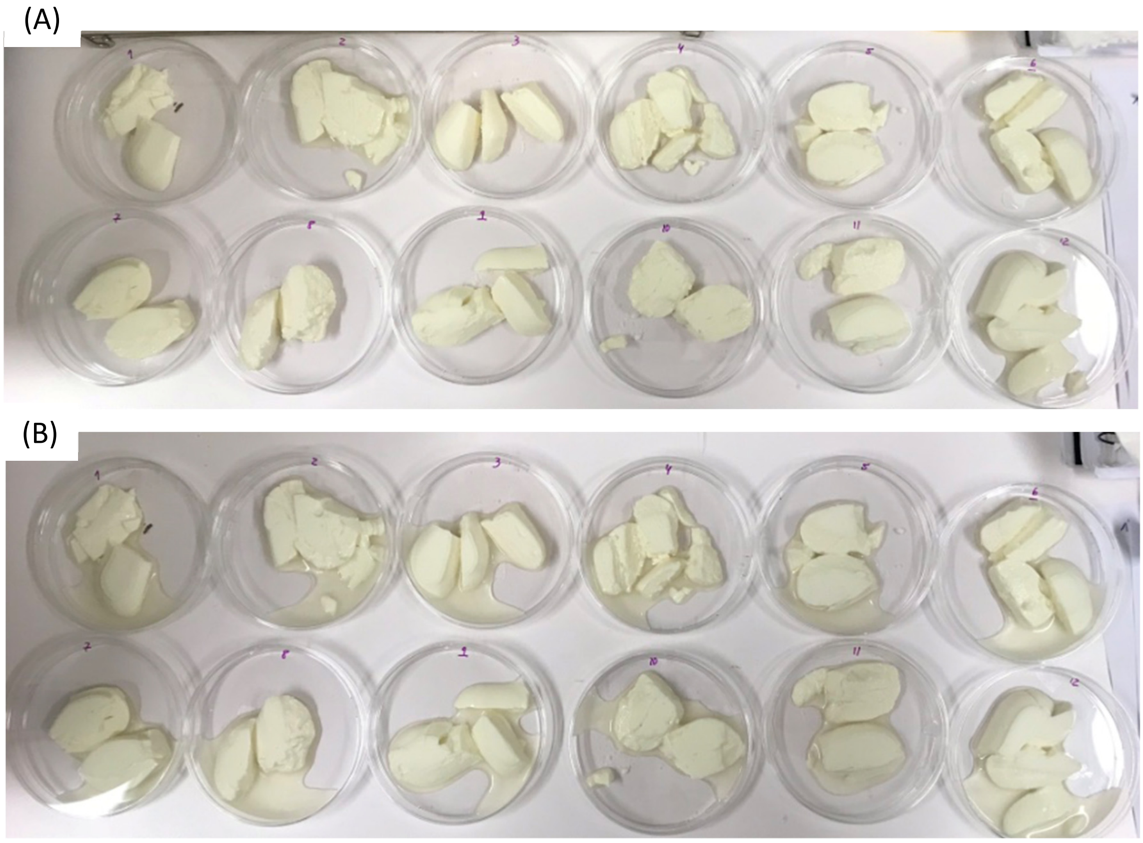

Figure S5. Surface Model: curds obtained from HPP milk pre-treated according to central composite design (**A**) 0 h after centrifugation and (**B**) after 2 h

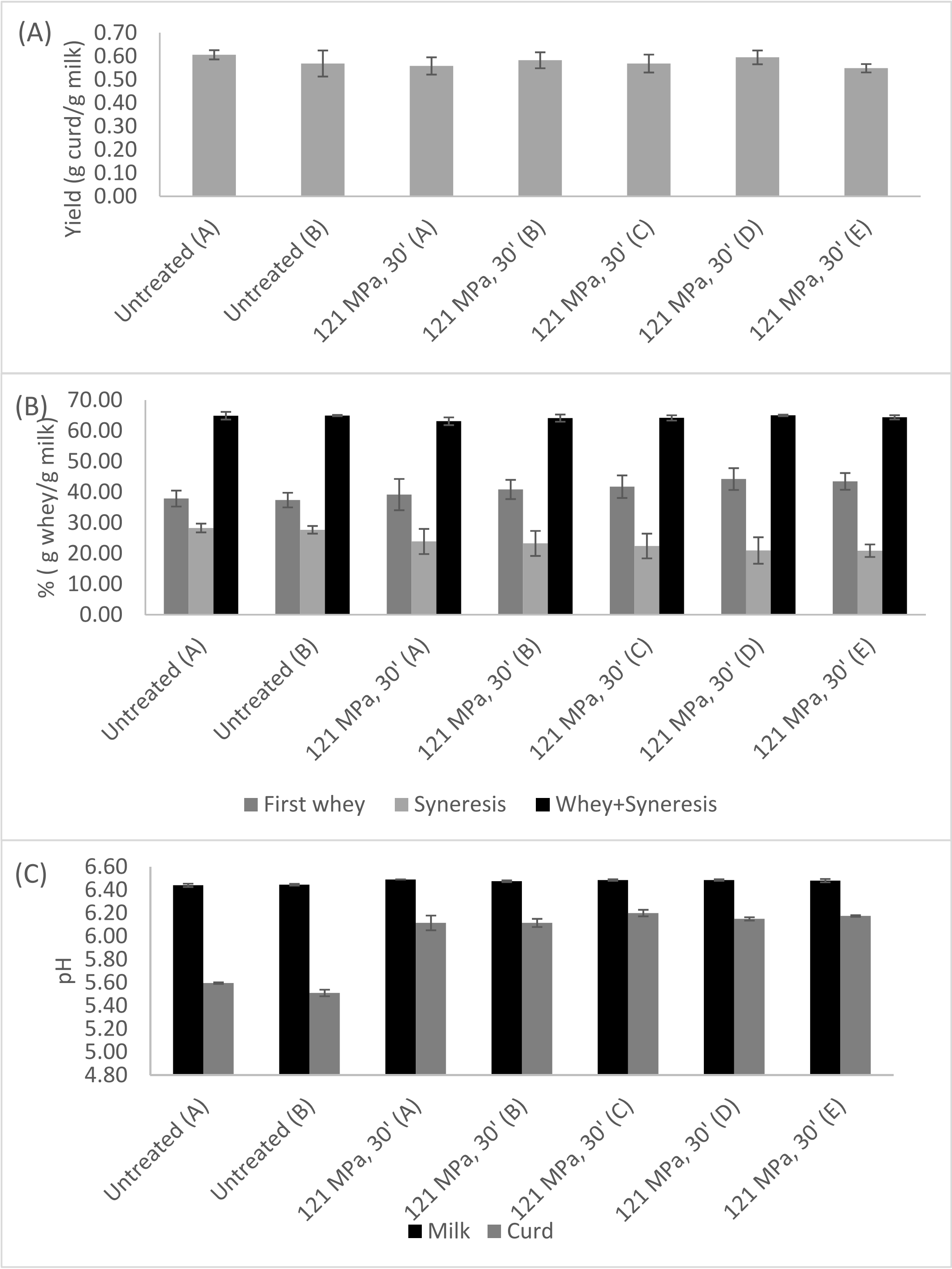

Figure S6. Validation model: (**A**) yield expressed in g of curd / g of milk; (**B**) whey release immediately after centrifugation: first whey; syneresis (24 h); whey+syneresis; (**C**) milk and curd pH-values from HPP milk pre-treated.
